# Choroidal Metastatic Carcinoma Accompanied With Sjögren Syndrome Initially Presenting as Acute Glaucoma With Angle Closure: Case Report

**DOI:** 10.3389/fopht.2022.751166

**Published:** 2022-03-17

**Authors:** Chunli Chen, Feng Hu, Yizhe Cheng, Zhixiang Hu, Ge Wang, Xiaoyan Peng

**Affiliations:** ^1^ Department of Ophthalmology, Beijing Tongren Hospital, Capital Medical University, Beijing, China; ^2^ Beijing Ophthalmology and Visual Science Key Laboratory, Capital Medical University, Beijing, China; ^3^ Eye Hospital, School of Ophthalmology and Optometry, Wenzhou Medical University, Wenzhou, China

**Keywords:** choroid metastatic carcinoma, Sjögren syndrome, angle-closure glaucoma, uveal effusion syndrome, case report

## Abstract

**Background:**

To report a case of choroidal metastatic carcinoma accompanied by Sjögren syndrome (SS) initially presenting as acute glaucoma with angle closure.

**Case Presentation:**

A 47-year-old woman complaining about swelling pain and blurred vision in the right eye for 3 days had a notable previous history of dry eyes, dry mouth, and joint pain. In another clinic, she was misdiagnosed as having acute glaucoma with angle closure, but she had poor response to eye drops and intravenous drip of mannitol for controlling intraocular pressure. The intraocular pressure in the right eye was 49 mm Hg, yet with clear cornea, shallow peripheral anterior chamber depth with 1/4 cornea thickness and fixed and dilated pupil. Macular folds were noted through a 90-D lens *via* slit lamp. Therefore, the diagnosis of secondary glaucoma was considered. Further examinations were conducted. Ultrawide-field fundus image showed retinal detachment with choroidal detachment in the right eye with suspected solid occupation of choroid metastatic cancer. B-scan ultrasound showed an elevated mass in the posterior pole of the ocular wall. The patient showed very good response to local corticosteroid eye drops after 3 days with deepening of the anterior chamber and significant decline of intraocular pressure. The brain, ocular magnetic resonance imaging, and lung computed tomography with enhancement showed lung cancer and choroidal metastatic carcinoma. Immunological abnormalities and symptoms supported the diagnosis of SS. After 1-month systematic chemotherapy and local–regional radiotherapy, retinal and choroidal detachment was restored with a stable intraocular pressure.

**Conclusion:**

The ophthalmologist should pay attention to differential diagnosis of angle-closure glaucoma from secondary glaucoma in cases with choroidal–retinal detachment or macular folds, which could be an ocular manifestation of choroidal metastatic carcinoma or SS in rare condition.

## Background

Choroid metastatic carcinoma is a rare condition with less than 1% frequency of all intraocular malignant tumors. The most common primary diseases of choroid metastatic carcinoma are lung and breast cancer (1, 2). Approximately 35% of patients with lung cancer were found to have extrapulmonary lesions earlier than primary pulmonary lesion (3). Five percent of choroidal metastatic carcinoma patients were diagnosed as having primary angle-closure glaucoma or secondary glaucoma initially (4). Sjögren syndrome (SS) is a chronic autoimmune rheumatic disease involving the exocrine gland, especially the lacrimal gland; thus, characteristic symptoms include dry eyes and mouth. SS could also initially present as uveal effusion syndrome (UES) in rare condition (5). Severe complications of SS include a range of malignant tumor. It is also reported that the incidence of cancer is higher in patients with SS than those not (6–8). Secondary glaucoma following SS combined with choroid metastatic carcinoma was not reported in the publications. We report this case, which aims to reveal the internal relapse and interaction of the diseases.

## Case Presentation

The case of a 47-year-old woman complaining about swelling pain and blurred vision in the right eye for 3 days is presented. Three days prior, the patient had swelling and pain accompanied by blurred vision, red eyes, and tears in the right eye without obvious incentive. She was diagnosed as having “acute angle-closure glaucoma in the right eye”; antiglaucoma medications and intravenous drip of mannitol were applied to control intraocular pressure (IOP). The symptoms were not relieved. She had binocular ametropia for more than 20 years, history of intermittent headache, ocular pain, dry eyes and mouth, lower backache, and joint pain for half a year. The patient denied smoking and occupational exposure.

**Figure 1:**
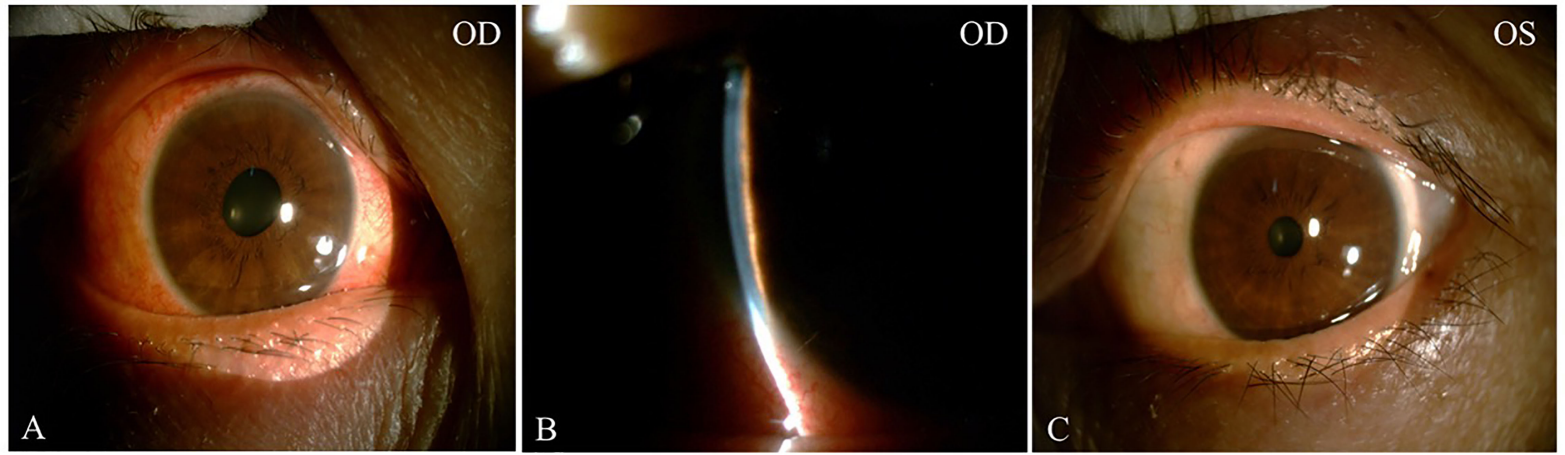
The external and slit-lamp photograph at initial presentation. External photograph showed ciliary congestion and fixed pupil with diameter of 4mm in the right eye **(A)**. Slit-lamp exams revealed extremely shallow anterior chamber with narrow chamber angle less than 1/4 cornea thickness **(B)**. The external photograph of the left eye was normal **(C)**.

**Figure 2:**
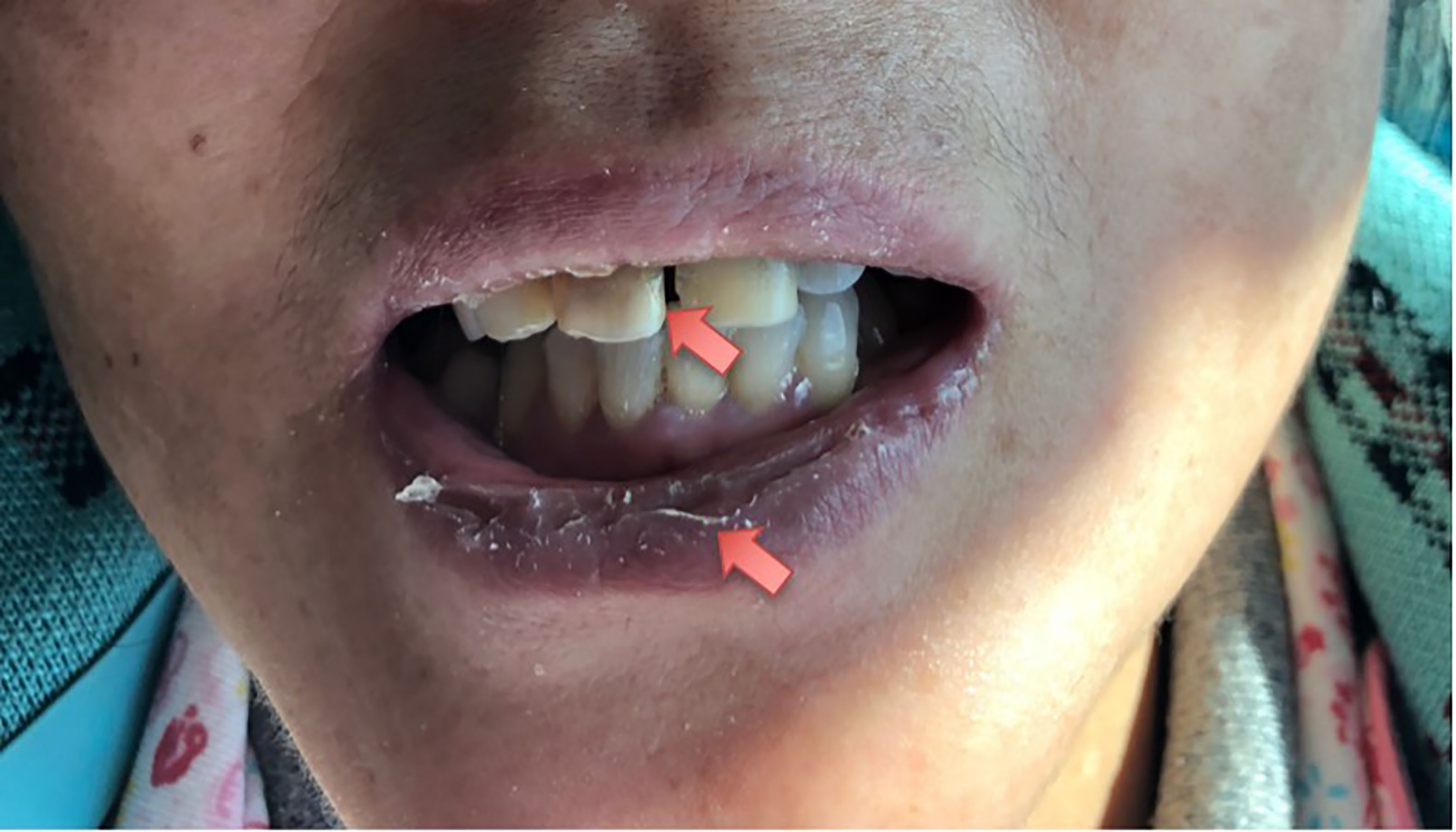
Local facial photograph of the patient. The lips of the patient were very dry, and there was dental caries on upper incisor teeth.

On initial examination, her best corrected visual acuity (BCVA) was 20/60 OD and 20/25 OS; refractive statues were −7.25/−1.25 × 125 in the right eye and −4.75/−0.75 × 115 in the left eye. IOP was 49 mm Hg OD, 18 mm Hg OS. Slit-lamp examinations revealed ciliary congestion, clear cornea, shallow anterior chamber with 1/4 cornea thickness of peripheral AC depth, fixed pupil with diameter of 4 mm, cell and flare (−) in the anterior chamber, and normal lens in the right eye (**Figure 1**). Ninety-diopter lens *via* slit lamp showed a clear optic disc edge with a cup–disc ratio of 0.3 and macular folds. There was no remarkable abnormality in the left eye. Gonioscopy showed angle closure in the right eye and open angle in the left eye. Tear break-up time was shorter than 10 s bilateral, and Schirmer score without anesthesia was less than 10 mm at 5 min bilateral. The lips of the patient were very dry, and there was dental caries on the upper incisor teeth (**Figure 2**).

**Figure 3:**
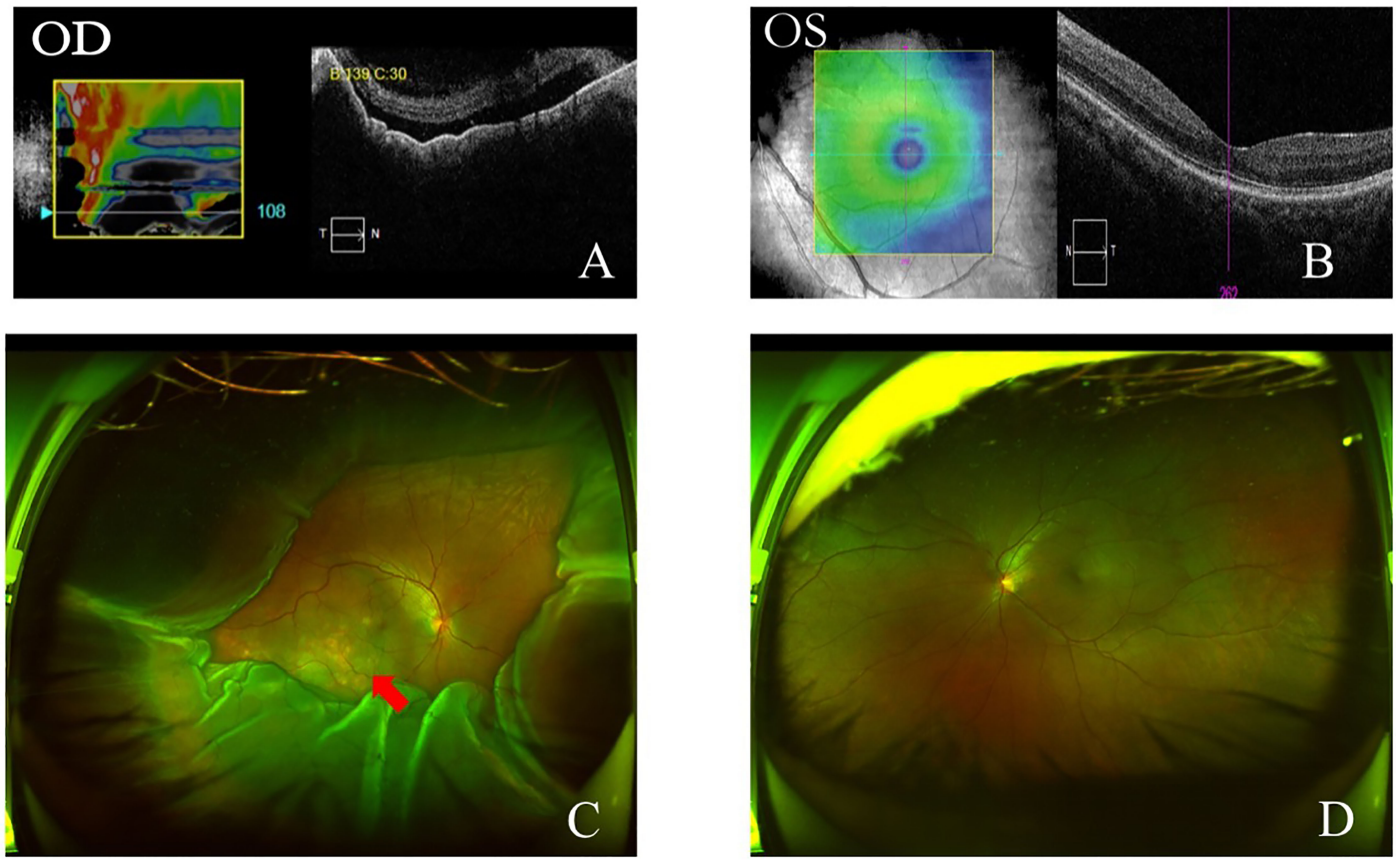
OCT and ultra-wide-field fundus image at initial presentation. OCT showed neuroepithelial retinal detachment in macular region in the right eye **(A)**. Macular area was normal on OCT in the left eye **(B)**. Ultra-wide-field fundus image showed choroidal and retinal detachment in the right eye, suspected solid occupation of choroid metastatic cancer (red arrow) **(C)**. Ultra-wide-field fundus image of the left eye was normal **(D)**.

**Figure 4:**
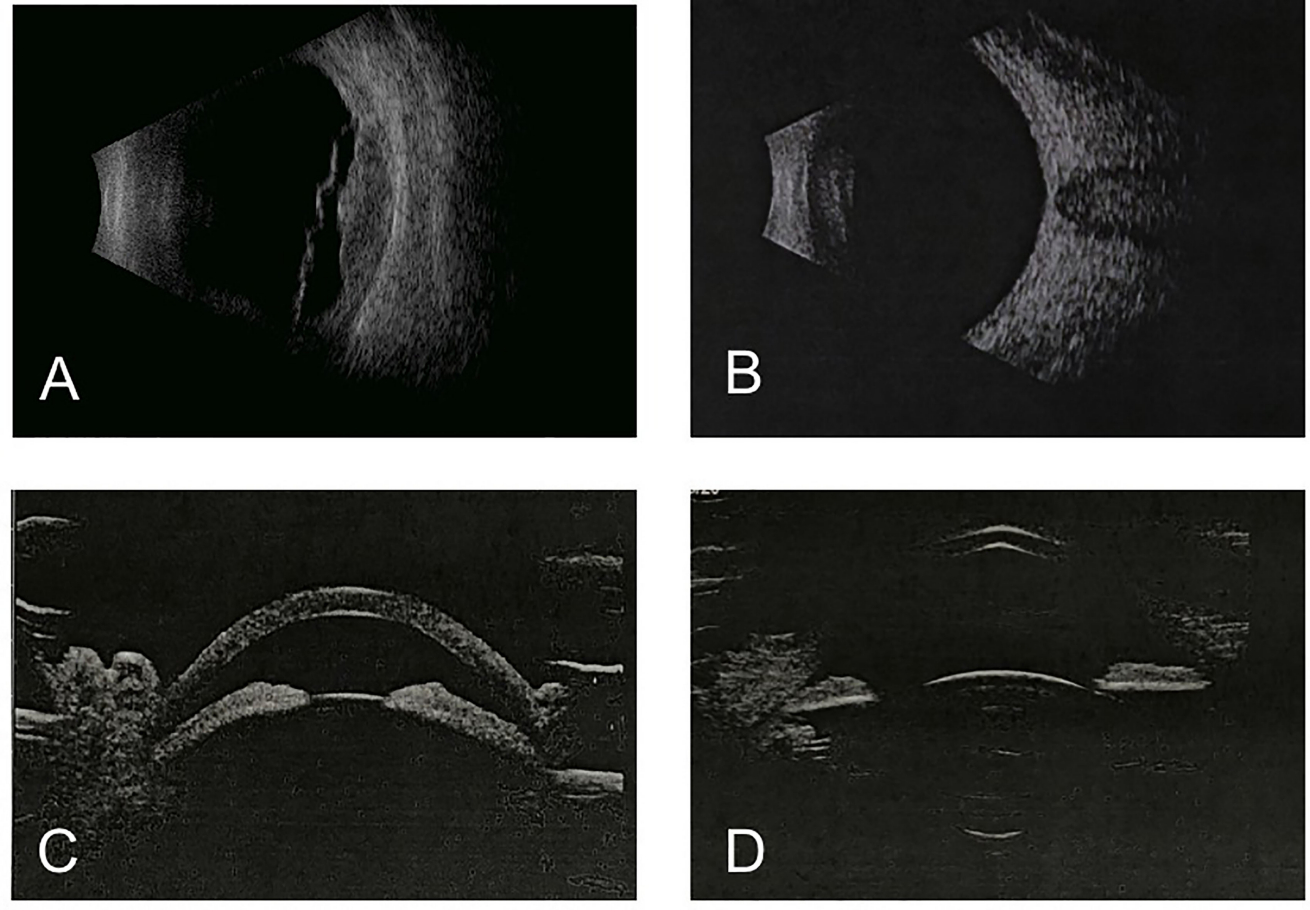
B-scan ultrasound and UBM at initial presentation. B ultrasound showed a elevated and homogeneous mass with intermediate-to-high echogenic sound and wavy notch **(A)**. B-scan ultrasound of the left eye was normal **(B)**. UBM showed shallow anterior chamber with lens-iris diaphragm moving forward **(C)**. UBM of the left eye was normal **(D)**.

**Figure 5:**
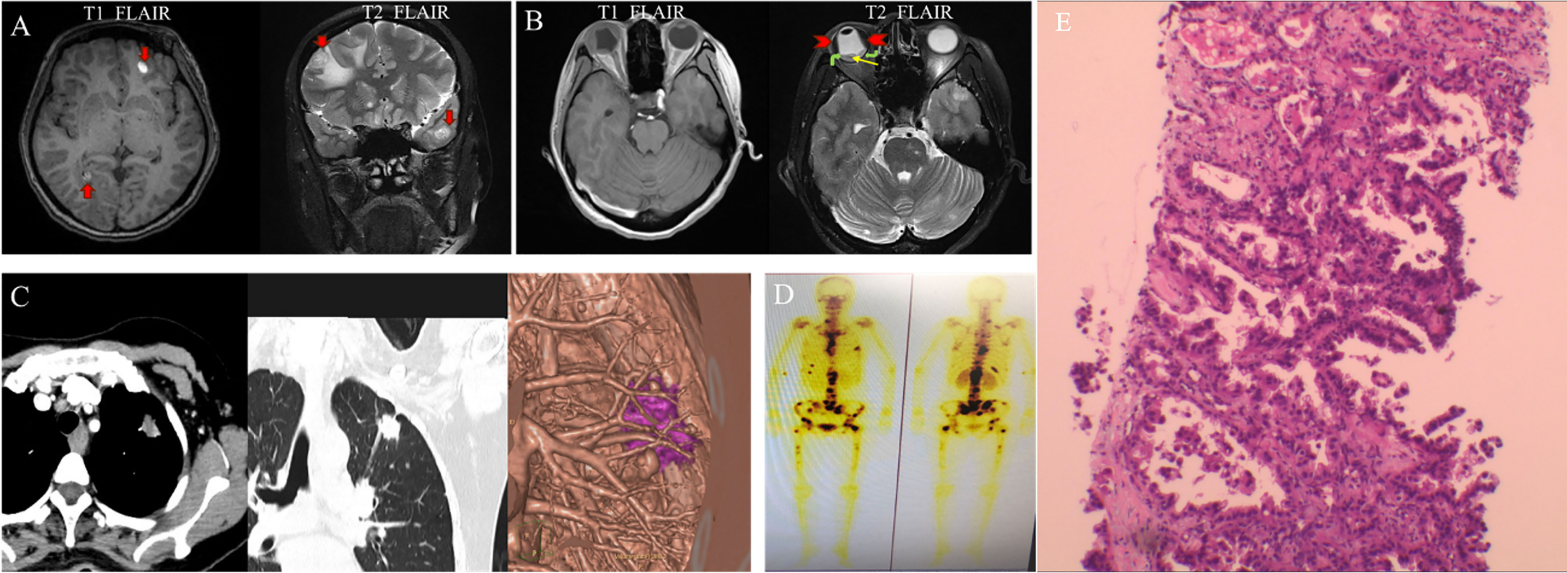
Brian MRI, lung CT and radionuclide exam at initial presentation. MRI T1 FLAIR showed oval high signal (red arrow), and T2 FLAIR showed oval high signal surround by low signal on the right frontal lobe and left temporal lobe (red arrow), which indicated mass and peripheral edema **(A)**. MRI T1 FLAIR showed slightly high signal inside the right eye, and T2 FLAIR showed low signal (red arrowhead) and low signal in band-shape surrounded by relatively high signal (yellow and green arrow) **(B)**. Pulmonary enhancement CT showed lobulated nodule with burrs and pleural traction **(C)**. Radionuclide exam showed strong radiological signal in vertebral column, ribs, and femur **(D)**. Pathological picture of the lung biopsy. The tumor cells showed moderate atypia, most tumor cells were arranged in acinar structure, and formed papillary hyperplasia into the lumen. The tumor cells grew adherent. The above growth patterns suggested infiltration. The maximum diameter of the invasive area was more than 5 mm, which consistent with invasive adenocarcinoma **(E)**.

Anterior segment optical coherence tomography (OCT) showed that the depths of anterior chamber were 1.29 mm OD and 2.58 mm OS. OCT showed neuroepithelial detachment in the macular area in the right eye and no remarkable abnormality in the left eye (**Figures 3A, B**). Ultrawide-field fundus image showed choroid retinal detachment in the right eye with suspected solid occupation of choroidal lesion (**Figures 3C, D**). The B-scan ultrasound showed an elevated and homogeneous mass with intermediate to high echogenic sound and wavy notch retinal and choroidal detachment, and hollowing-out sign and choroidal excavation were not found in the right eye. There was no obvious abnormality in the left eye (**Figures 4A, B**). Ultrasound biomicroscope (UBM) showed that the anterior chamber of the right eye was shallow with lens-iris diaphragm moving forward, and no ciliary body mass or supraciliary effusion was found (**Figures 4C, D**). Brain magnetic resonance imaging (MRI) showed multiple lesions in the brain with hemorrhage inside (**Figure 5A**), subretinal fluid (suspected hemorrhage), and a mass signal in the posterior segment of the right eye (**Figure 5B**). Immunological infection–related tests were positive for antinuclear antibody (ANA) (1:100) and anti-SS-A/Ro-52KD antibody; ANA (1:1,000). Computed tomography (CT) scan showed a high-density lobulated mass (2.4 × 1.7 cm on cross-section scanning) with irregular burrs and pleural traction in the upper lobe of the left lung, which showed inhomogeneous enhancement. There were multiple high-density nodules in bilateral lung, and multiple enlarged lymph nodes were significantly enhanced in the left supraclavicular, mediastinal, left hilar area (**Figure 5C**). Radionuclide examination revealed abnormally strong radiological signal in multiple bones involving the vertebral column, ribs, and femur (**Figure 5D**). CT-guided lung biopsy was performed, and diagnosis of invasive adenocarcinoma (left upper lobe) was proven (**Figure 5E**).

**Figure 6:**
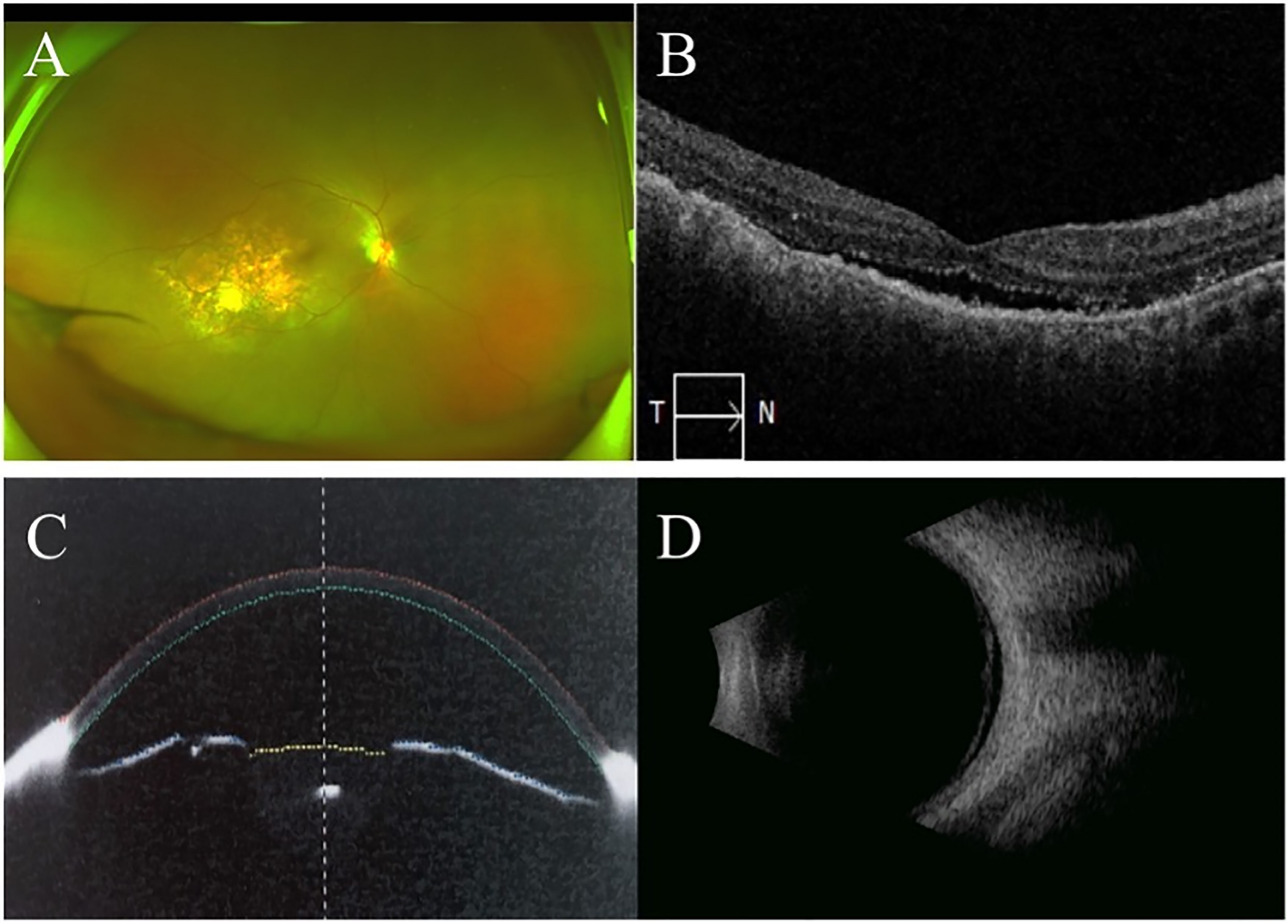
Ultra-wide-filed fundus image, posterior and anterior OCT, B-scan ultrasound one month after radiological therapy. Ultra-wide-filed fundus image showed subretinal white lesions inside vascular arch, relief of retinal and choroidal detachment **(A)**. OCT showed few subretinal fluid and small nodules on RPE in the macular area in the right eye **(B)**. Anterior OCT showed increase of anterior chamber depth in the right eye **(C)**. B-scan ultrasonic image of the right eye showed relief of retinal and choroidal detachment **(D)**.

After consultation with the rheumatology and oncology departments, diagnosis of secondary glaucoma and choroidal metastasis carcinoma in the right eye, primary lung invasive adenocarcinoma, and multiple metastases (brain, lymph node, bone) with primary SS was made. Besides brinzolamide and timolol maleate, tobramycin dexamethasone eye drops were also locally applied to the right eye. IOP decreased to normal range 3 days after treatment. After obtaining informed consent from the patient and her family, the patient underwent systemic chemotherapy (pemetrexed 0.9 dL and gefitinib 250 mg once a day), and choroid and cranial metastasis lesions underwent radiotherapy (2F-CRT: eye 4,000 OcGy/20f; brain 4,000 OcGy) because of the localized quality of the lesions. After 1-month systematic chemotherapy and local–regional radiotherapy combined with medications for controlling IOP and anti-inflammation, BCVA improved to 20/50 OD; refractive status switched from −7.25/−1.25 × 125 to −5.25/−1.25 × 125. IOP was within normal range in both eyes. Pupil remained fixed with a diameter of 4 mm in the right eye. Lens was normal, and there were subretinal white lesions inside vascular arch (**Figure 6A**). OCT showed few subretinal fluid and small nodules on retinal pigment epithelium (RPE) in the macular area of the right eye (**Figure 6B**). The depth of anterior chamber improved to 2.39 mm in the right eye (**Figure 6C**). B-ultrasound showed relief of retinal and choroidal detachment in the right eye 1 month after therapy (**Figure 6D**). Further ocular examinations for follow-up could not be applied because she was in bed all the time. Unfortunately, this patient died 1½ years after diagnosis of lung-invasive adenocarcinoma and multiple metastases.

## Discussion

Uveal metastatic carcinoma is a malignant tumor that spreads through blood circulation from other parts of the body. The most common location of uveal metastatic carcinoma is choroid (88%) (1). The primary lesions are mainly lung cancer and breast cancer. Choroid metastatic carcinoma is mostly seen in patients aged 40 to 70 years old, and the fundus presented as round or irregular yellowish white or grayish yellow flat prominent lesions in the posterior pole, and a few were flat hemispherical prominent masses. Among them, 75% are uniocularly involved; fundus lesions can be isolated or multiple (2, 6, 9, 10). Most patients with choroidal metastatic carcinoma presented with visual loss due to the direct invasion of carcinoma in the macular area and exudative retinal detachment. Previous study showed that 5% of choroid metastatic carcinomas were diagnosed as primary angle-closure glaucoma or secondary glaucoma (4).

SS is a chronic systemic autoimmune disorder, with characteristic presentation of dry eye and mouth, including primary and secondary SS (11, 12). We previously reported a series of primary SS, which initially presents as uveal suffusion syndrome, optic neuritis, neurologic disorders, and pulmonary nodules (5). A previous study showed that the incidence of lung cancer in primary SS was 0.477%, which was significantly higher compared with those without SS. Consensus criteria for primary SS are as follows: anti-SSA (Ro)+ (weight 3), biopsy of salivary gland showing focal lymphocytic sialadenitis with a focus score ≥1/4-mm^2^ tissue surface area (weight 3), and assessment of dry eye or mouth (weight 1) (13). Symptoms of dry eye and mouth and positive anti-SSA supported the diagnosis of SS in the present case.

Secondary glaucoma was diagnosed in this case, with increased IOP in the right eye and normal IOP in the left eye with deep anterior chamber. The mechanism of secondary glaucoma was well known (14). For the present case, acute attack of glaucoma may be associated with exacerbation of intraocular inflammation; there were three clues: first, she complained about long-term chronic ocular pain, which indicated intraocular ocular inflammation. Second, good response to tobramycin dexamethasone eye drops for reducing IOP supported intraocular inflammation. Third, her MRI indicated subretinal hemorrhage, but there was no subretinal hemorrhage on fundus image, so subretinal hemorrhage signal on MRI may reflect lots of inflammatory cells in the subretinal fluid. Besides, spontaneous necrosis of choroidal metastatic carcinoma due to insufficient blood supply with growth of tumor may lead to secondary inflammation. Choroidal metastatic carcinoma may also cause ischemia, necrosis, and inflammation by compressing normal ocular tissue (15). Considering that the choroid metastases lesions were not big enough to induce such a serious retinal detachment with choroidal detachment, we speculated that there was overlap in process of UES and SS, and immunological factors play an important role in these two disorders (5). However, the exact immune mechanism and signal pathways are still unclear and need further investigation in UES and SS. Therefore, for the present case, the acute attack of glaucoma and angle closure may be associated with sudden increase in posterior IOP due to sudden exacerbation of inflammation and liquid exudation secondary to choroidal tumor and SS. Exudative retinal detachment may be associated with choroidal vascular damage and impaired integrity of RPE, which are caused by invasion and enlargement of choroidal metastatic tumor.

Treatment of choroidal metastases includes systemic and topical therapy (16–19). The choroidal metastasis of lung cancer belongs to the terminal stage of the tumor, and the systemic chemotherapy is the first choice for treatment. At present, radiotherapy is the first choice for metastatic choroidal carcinoma (20). For the present case, craniocerebral lesions and ocular choroidal metastases were treated with radiotherapy. The radiology therapy was effective with serological tumor markers decreasing 2 weeks later and improvement of BCVA and relief of retinal and choroidal detachment 1 month later.

In conclusion, choroid metastatic carcinoma may occur before the primary cancer diagnosis and may initially present as acute angle-closure glaucoma. The ophthalmologist should pay attention to the differential diagnosis of angle-closure glaucoma from secondary glaucoma in cases with choroidal–retinal detachment or macular folds, which could be an ocular manifestation of choroidal metastatic carcinoma in rare condition. The UES may be induced by choroid metastatic carcinoma and SS jointly. It is noteworthy that the pathogenesis of UES is complicated, and comprehensive ocular and laboratory examinations are necessary in patients with factors suggestive of the disease. Enhanced CT scan and radiological examination are helpful for the detection of primary and metastatic lesions.

## Data Availability Statement

The original contributions presented in the study are included in the article/supplementary material. Further inquiries can be directed to the corresponding author.

## Ethics Statement

Ethical review and approval was not required for the study on human participants in accordance with the local legislation and institutional requirements. Full verbal and written consent have been obtained from the patient for submission of this manuscript for publication.

## Author Contributions

Writing: FH. Draft preparation: CC. Conceptualization: ZH and GW. Review and editing: XP. All authors have read and approved the manuscript.

## Funding

Thanks to support by The Capital Health Research and Development of Special (No.SF-2018-2-1081), Capital Medical University Affiliated Beijing Tongren Hospital Key Medical Development Plan (trzdyxzy201801). The funding organization had no role in the design or conduct of this research.

## Conflict of Interest

The authors declare that the research was conducted in the absence of any commercial or financial relationships that could be construed as a potential conflict of interest.

## Publisher’s Note

All claims expressed in this article are solely those of the authors and do not necessarily represent those of their affiliated organizations, or those of the publisher, the editors and the reviewers. Any product that may be evaluated in this article, or claim that may be made by its manufacturer, is not guaranteed or endorsed by the publisher.

## Abbreviations

BCVA, best corrected visual acuity; IOP, intraocular pressure; UBM, ultrasound biomicroscope; ANA, antinuclear antibody; SS, Sjögren syndrome; UES, uveal effusion syndrome.
